# Suppression of sonic hedgehog pathway-based proliferation in glioblastoma cells by small-size silver nanoparticles in vitro

**DOI:** 10.1007/s00204-023-03552-x

**Published:** 2023-07-05

**Authors:** Bartosz Skóra, Martyna Masicz, Patrycja Nowak, Jagoda Lachowska, Paulina Sołtysek, Justyna Biskup, Paulina Matuszewska, Konrad A. Szychowski

**Affiliations:** 1 Department of Biotechnology and Cell Biology, Medical College, University of Information Technology and Management in Rzeszow, St. Sucharskiego 2, 35-225 Rzeszow, Poland; 2 Medical Biotechnology Student’s Science Group “Helisa”, Medical College, University of Information Technology and Management, St. Sucharskiego 2, 35-225 Rzeszow, Poland

**Keywords:** Silver nanoparticles, Gliomas, Sonic hedgehog pathway, Cancer proliferation, Reactive oxygen species

## Abstract

Glioblastomas (GBs) are one of the most aggressive and invasive intracranial cancers. Recently, it has been postulated that, among other factors, the hedgehog (HH) pathway may be a key factor in this phenomenon. Moreover, it has been reported that small-size silver nanoparticles (AgNPs) are characterized by a high cytotoxic effect towards GBs. However, their effect on the sonic hedgehog (SHH) pathway has never been demonstrated in any cancer cells. Therefore, the aim of the present study was to evaluate the impact of the anti-proliferative properties of 5-nm AgNPs on the SHH pathway in the GB cell line (U-87MG) in vitro*.* The results showed a time- and dose-dependent decrease in the metabolic activity in the U-87MG cells treated with AgNPs, with IC_50_ reaching 30.41 and 21.16 µg/mL after 24 h and 48 h, respectively, followed by an increase in the intracellular reactive oxygen species (ROS) level. The co-treatment of the cells with AgNPs and Robotnikinin (SHH inhibitor) abolished and/or strengthened the effect of AgNPs, especially on the *SHH* mRNA levels and on the PCNA, PTCH1, Gli1, and SUFU protein levels. Interestingly, no changes in the level of ERK1/2, Akt, and SRC kinase protein expression were detected, suggesting a direct impact of AgNPs and/or ROS on the inhibition of the canonical SHH pathway. However, more studies are needed due to the increase in the mTOR protein expression after the treatment of the cells with AgNPs, as in the Robotnikinin treatment. In conclusion, small-size AgNPs are able to inhibit the proliferation of GB cells in vitro by suppressing the canonical SHH pathway.

## Introduction

Glioblastomas (GBs) are glioma-derived cancers with a poor survival rate, estimated at approx. 1 year. Moreover, according to the World Health Organization (WHO), GBs are responsible for 2% of all the intracranial tumors nowadays. This phenomenon is linked with many aspects, such as the high selectivity of the blood–brain barrier (BBB) or the presence of the skull (Du et al. 2021; Li et al. 2016a). Moreover, GBs are characterized by high malignancy with enormous proliferation and migration potential resulting in the invasiveness of such cancer cells (Kim 2013). Among many pro-proliferative pathways in GBs, literature data show the pivotal role of the phosphoinositide 3-kinase/protein kinase B (PI3K/Akt), Janus kinase-signal transducer and activator of transcription (JAK-STAT), and mitogen-activated protein kinase/extracellular signal-regulated kinase (MAPK/ERK) pathways as key factors in the adhesive and invasive properties of such cancer cells (Ou et al. 2021; Ramaswamy et al. 2019; Li et al. 2016b). Although new anti-cancer strategies based on the inhibition of the above-mentioned pathways are being developed, the results obtained also in clinical trials are not satisfactory (Li et al. 2016b). As shown *inter alia* by Day et al., GBs are able to bypass the blocked proliferation-related pathways, which is one of the causes of the insufficiency of novel anti-cancer approaches (Day et al. 2020). Interestingly, the hedgehog pathway (HH) has recently been identified as another key factor in the aggressiveness of GBs, as described *inter alia* by Hung et al. (Hung et al. 2020).

HH is a conservative intracellular pathway, which is crucial in embryo development, cell division, and proliferation processes (Carballo et al. 2018). Physiologically, three activating ligands of HH have been described—sonic hedgehog (SHH), Indian hedgehog (IHH), and desert hedgehog (DHH). They are able to abolish the inhibitory effect of patched 1 and/or 2 (PTCH1/2) transmembrane proteins, which leads to activation of the smoothened (SMO) protein (Gergues et al. 2021). Subsequently, the signal is transduced intracellularly (with engagement of the SUFU protein), resulting in the activation of the zinc finger protein (Gli1), a well-described oncogene, which stimulates cells to proliferate (Zhu and Lo 2010). Interestingly, alterations in the HH pathway have been linked with development of high-grade GBs as well as increased cancer invasiveness (Takezaki et al. 2011; Wang et al. 2022). Many efforts have been made to use the HH pathway as a target in the anti-GB treatment, e.g., the inhibition of the activity of the SMO protein, which was shown by Bissey et al. to have failed during clinical trials (Bissey et al. 2020). This phenomenon has been linked with the overexpression of HH ligands, mainly SHH, by GBs, which is based on the auto- and paracrine regulation abolishing the HH inhibition at the SMO level (Bissey et al. 2020). Interestingly, a study has linked the ability of silver nanoparticles (AgNPs) to suppress the ATP-binding transporter (ABC), whose over-activation is a well-established feature of the multidrug-resistance (MDR) in cancer cells (Kovács et al. 2016).

In accordance with the definition used worldwide, AgNPs are structures with a size ranging between 1 to 100 nm characterized by high reactivity and internalization into the cell, resulting from their shape and size (Perde-Schrepler et al. 2019). AgNPs are usually described as pro-oxidative factors with an ability to impair the *redox* homeostasis by releasing high amounts of silver ions (Ag^+^), which in consequence leads to oxidative stress and apoptosis (Ullah et al. 2020). Interestingly, this phenomenon has been observed in both normal and cancer cells in vitro. However, Liang et al. have shown that AgNPs can act as potential sensitizers in temozolomide-resistant glioma cells (U-251) in vitro (Liang et al. 2017). Similarly, Liu et al. have proved that AgNPs enhance the anti-proliferative effect of radiotherapy in the U-251 glioma cell line (Liu et al. 2018a). Moreover, the toxic effect of these NPs has been reported repeatedly *inter alia* in human breast, lung, tongue, skin, brain, and hepatic cancer cell lines in vitro (Perde-Schrepler et al. 2019; Skóra et al. 2022, 2023; Hepokur et al. 2019; Faedmaleki et al. 2014*).* The aforementioned conclusions are promising, given the postulated ability of AgNPs to cross the BBB (Dan et al. 2015; Khan et al. 2019). However, none of the studies have determined the interaction between these NPs and the HH pathway, which is crucial due to its potential engagement in the failure of the current anti-cancer treatment. Additionally, human U-87MG cells are frequently used as a model for testing the anti-cancer properties of AgNPs in GBs due to *inter alia* their aggressiveness and proliferation properties; hence, these cells were chosen as a model in the present study (Louca et al. 2019).

The aim of this study was to determine the impact of 5-nm AgNPs on the SHH pathway in a glioblastoma cell line (U-87MG) in vitro*.* The SHH and its role in the anti-proliferative properties of AgNPs in this type of cancer was assessed as well as the potential mechanism of action of such NPs in comparison to Robotnikinin (a selective inhibitor of the SHH pathway). The metabolic activity, intracellular ROS level, and mRNA and protein expression were determined to understand the role of the SHH pathway in the AgNP cytotoxicity potential in GBs.

## Materials and methods

### Reagents

Phosphate buffer saline (PBS) and Dulbecco's Modified Eagle Medium (DMEM) were purchased from Corning (Corning, USA). Trypsin, resazurin sodium salt penicillin,, 2′,7′-dichlorodihydrofluorescein diacetate (H_2_DCF-DA), streptomycin, ethanol, ammonium persulfate (APS), methanol, N,N,N′,N′-tetramethyl ethylenediamine (TEMED), acrylamide/bisacrylamide, 30%, 37.5:1 ratio, glycine, Tris Base, Tris–HCl, sodium chloride (NaCl), silver nanoparticles (PVP-stabilized) with a small size (5 nm)—AgNPs, bovine serum albumin (BSA), sodium dodecyl sulfate (SDS) and Bradford reagent were purchased from Merck (Darmstadt, Germany). The High-Capacity cDNA Reverse Transcription Kit, Restore™ Western Blot Stripping Buffer, primers and TaqMan® probes complementary to sequences, encoding the *GAPDH (Hs02758991_g1)* and *SHH (Hs00179843_m1),* and goat anti-mouse and goat anti-rabbit HRP-conjugated antibodies were obtained from ThermoFisher (Waltham, USA). Fetal bovine serum (FBS), Fast Probe qPCR Master Mix (2x), Universal RNA Purification Kit, Perfect Tricolor Protein Ladder, and RIPA buffer were purchased from EURx (Gdańsk, Poland). The PVDF membrane, anti-GAPDH, anti-CAT1, and anti-SOD1 mouse primary antibodies were purchased from Santa Cruz Biotechnology (Santa Cruz, USA). Anti-PTCH1, anti-Gli1, anti-SHH, anti-SUFU, anti-Akt, anti-mTOR, anti-SRC, anti-ERK1/2, and anti-PCNA primary antibodies were obtained from ABClonal (Düsseldorf, Germany). Robotnikinin (cat. 13204) was purchased from Cayman Chemicals (Ann Arbor, USA). The PVP-stabilized AgNPs were chosen based on their high stability and low aggregation properties in aqueous solutions, comparing to, e.g., citrate-stabilized AgNPs proved previously in the literature report (Tejamaya et al. 2012).

### Cell culture and treatment

The glioblastoma cell line (U-87MG, HTB-14™) was purchased from the American Type Culture Collection (ATCC). Cells were grown in DMEM with 10% FBS supplemented with 0.1% penicillin/streptomycin at 37℃ with 5% CO_2_ until reaching confluency. Afterwards, the U-87MG cells were trypsinized and seeded on 96-well plates (for resazurin reduction and H_2_DCF-DA assays), 12-well plates (for RT-qPCR), and a ⌀100 mm culture dish (for Western Blot) at the density of 4 × 10^3^ cell/well, 1.2 × 10^5^ cell/well, or 2.2 × 10^6^ cell/well, respectively. Next, the cells were treated with increasing concentrations of AgNPs in a concentration range from 1 ng/mL to 100 µg/mL for 24 h and 48 h. Moreover, in the case of the RT-qPCR and Western Blot methods, the cells were treated with effective but non-lethal concentrations of the tested compounds (chosen based on the dose–response assays), namely 1 µg/mL of AgNPs and 10 µM of Robotnikinin for 6 h and 24 h (for RT-qPCR) or 24 h and 48 h (for Western Blot).

### Resazurin reduction assay

The assay was performed as described by Skóra et al. w/o modifications (Skóra et al. 2023). After the 24-h and 48-h treatment of the U-87MG cells with certain compounds (as described in subsection Cell culture and treatment), the measurement of fluorescence was performed at respective excitation and emission wavelength, using a microplate reader (FilterMax F5).

### Intracellular ROS level

The ROS level was quantified using 2′,7′-dichlorodihydrofluorescein diacetate (H_2_DCF-DA) as in Piechowiak et al. w/o modifications (Piechowiak et al. 2021). The measurement of fluorescence was performed at respective excitation and emission wavelength using a microplate reader (FilterMax F5).

### Confocal microscopy

The Calcein-AM and Hoechst 33342 staining was used in this study to determine the ability of tested AgNPs to affect the morphology of cell and nucleus (e.g., apoptotic-like) in tested U-87MG cells. The method was performed according to Skóra et al. (Skóra et al. 2023). The cells were seeded on a culture dish (⌀35 mm) at the density of 1 × 10^5^ cells/dish and sub-cultured for 24 h. Subsequently, the medium was exchanged to fresh one containing 1 µg/mL or 100 µg/mL of AgNPs. After 48 h, the cells were washed twice with warm PBS and the staining medium (serum-free), containing the 10 µM of Hoechst 33342, and 10 µM of Calcein-AM was applied for few min. Afterwards, fluorescence-based visualization was performed at certain excitation and emission wavelengths using a confocal microscope with a laser scanning module (ZEISS LSM700).

### RT-qPCR

The RT-qPCR analysis was performed as in Szychowski et al. w/o modifications (Szychowski and Gmiński 2019). 1 µg/mL of AgNPs, 10 µM of Robotnikinin, or 1 µg/mL of AgNPs and 10 µM of Robotnikinin were applied in the experiment for 6 h (primary gene response) and 24 h (secondary gene response). Primers and TaqMan® probes, specific for sequences, encoding the *GAPDH* and *SHH* genes were applied. The results were expressed as an average fold (avg. fold) calculated based on the ΔΔ_Ct_ obtained using the threshold value (Ct) for each sample during the exponential phase of the analyzed genes. *GAPDH* was used as a reference gene.

### Western Blot

The Western Blot analysis was performed as in Skóra et al. without modifications (Skóra et al. 2023). 1 µg/mL of AgNPs, 10 µM of Robotnikinin, or 1 µg/mL of AgNPs and 10 µM of Robotnikinin were applied in the experiment for 24 h and 48 h. The specific primary and secondary HRP-conjugated antibodies were used in the dilutions described in the table below (Table 1). Moreover, the Restore™ Western Blot Stripping Buffer was used as described by the producer to reprobe the PVDF membranes with other primary antibodies with minor modifications (ThermoFisher). Briefly, after the enhanced chemiluminescence-based detection, the membranes were washed once with TBS for 5 min with shaking, followed by 20 min. incubation at 45 °C with shaking in the aforementioned stripping buffer. Subsequently, the buffer was removed, and the membrane was washed three times with TBS at RT for 5 min, followed by 1-h non-specific side blocking with 1% of BSA in TBST. Next, the blocking solution was removed, and the membrane was reprobed with another primary antibody o/n at 4 °C. The detection was performed using the secondary HRP-conjugated antibodies as described in the above-cited paper.

**Table 1:** Types, catalog numbers, producers, and concentrations of primary and secondary antibodies used in the Western Blot method

Primary antibodies			HRP-conjugated antibodies		
Antibody target (host species)	Cat. number/producer	Dilution	Antibody target (host species)	Cat. number/producer	Dilution
anti-SOD1 (Mo)	sc-293226/SCTB	1:800	anti-Mo-HRP (Go)	#31430/ThermoFisher	1:10,000
anti-CAT1 (Mo)	sc-101523/SCTB	1:800			
anti-PTCH1 (Rb)	A0826/ABClonal	1:1000	anti-Rb-HRP (Go)	SH253595/ThermoFisher	1:10,000
anti-Gli1 (Rb)	A8387/ABClonal	1:1000			
anti-SHH (Rb)	A12695/ABClonal	1:1000			
anti-SUFU (Rb)	A6757/ABClonal	1:1000			
anti-Akt (Rb)	A18675/ABClonal	1:5000			
anti-SRC (Rb)	A19119/ABClonal	1:2000			
anti-ERK1/2 (Rb)	A16686/ABClonal	1:2000			
anti-PCNA (Rb)	A12427/ABClonal	1:2000			
anti-GAPDH (Mo)	sc-47724/ SantaCruz Bt	1:1000	anti-Mo-HRP (Go)	#31430/ThermoFisher	1:20,000

*Mo* mouse, *Rb* rabbit, *Go* goat, *HRP* horseradish peroxidase, *SCTB* Santa Cruz Biotechnology

### Statistical analyses

The data were expressed as means ± SD (standard deviations) of six (*n* = 6) or three (*n* = 3) repetitions of the experiments (specified in the caption of the graphs). The data were then used in the one-way analysis of variance (ANOVA) with Tukey’s post hoc test and denoted as *, **, *** for *p* < 0.05, *p* < 0.001, or *p* < 0.001, respectively, compared to control cells. The means denoted as # or *$* were statistically different at *p* < 0.05 between certain groups (marked on the graphs).

## Results

### Metabolic activity and ROS production

**Fig. 1:**
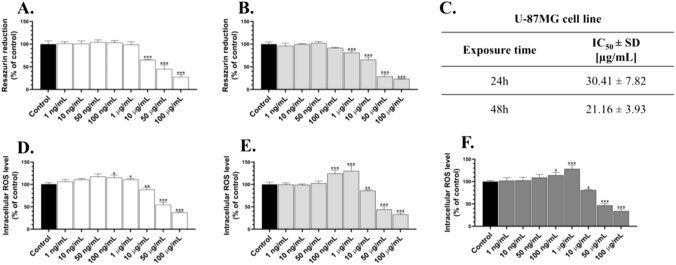
Metabolic activity (**A**–**B**) and the ROS production level (**D**–**F**) in the U-87MG cells treated with AgNPs in the concentration range from 1 ng/mL to 100 µg/mL for 6 h (**D**), 24 h (**A**, **E**), and 48 h (**B**, **F**). The calculated IC50 values for the respective time treatments shown in the right panel (**C**) were calculated based on the resazurin reduction measurement results. The means ± SD denoted as *, **, and *** are statistically different from the control at *p* < 0.05, *p* < 0.001, and *p* < 0.001, respectively

The U-87MG cells treated with 10 µg/mL, 50 µg/mL, and 100 µg/mL of AgNPs for 24 h were characterized by a 34.75%, 54.96%, and 72.44% decrease in the metabolic activity, respectively, compared to the control (Fig. 1A). In turn, after the treatment of the cells with 1 µg/mL, 10 µg/mL, 50 µg/mL, and 100 µg/mL of AgNPs, the metabolic activity decreased by 18.74%, 34.35%, 71.12%, and 76.38%, respectively, compared to the control (Fig. 1B). Similarly, the IC_50_ values increased during the time of the AgNPs’ exposure, reaching 30.41 µg/mL and 21.16 µg/mL after 24 h and 48 h, respectively (Fig. 1C).

After the 6-h treatment of the U-87MG cells with 100 ng/mL and 1 µg/mL of AgNPs, a 15.56% and 10.73% increase in the ROS production was observed, respectively, compared to the control, while the 10 µg/mL, 50 µg/mL, and 100 µg/mL concentrations of these NPs decreased the ROS level by 11.74%, 45.34%, and 62.60%, respectively, compared to the control (Fig. 1D). After the 24-h treatment, the ROS production in the cells treated with 100 ng/mL and 1 µg/mL of AgNPs increased by 24.56% and 30.19%, respectively, compared to the control (Fig. 1E). In turn, the 10 µg/mL, 50 µg/mL, and 100 µg/mL concentrations of AgNPs induced a 13.79%, 55.81%, and 66.90% decrease in this parameter in the U-87MG cells, respectively, compared to the control (Fig. 1E). Similarly, the cells treated with 100 ng/mL and 1 µg/mL of AgNPs for 48 h were characterized by a 14.22% and 28.21% increase in the intracellular ROS level, respectively, compared to the control (Fig. 1E). On the other hand, a 19.04%, 53.14%, and 66.01% decrease in this parameter was observed, compared to the control, respectively (Fig. 1E).

### Co-treatment of cells

**Fig. 2:**
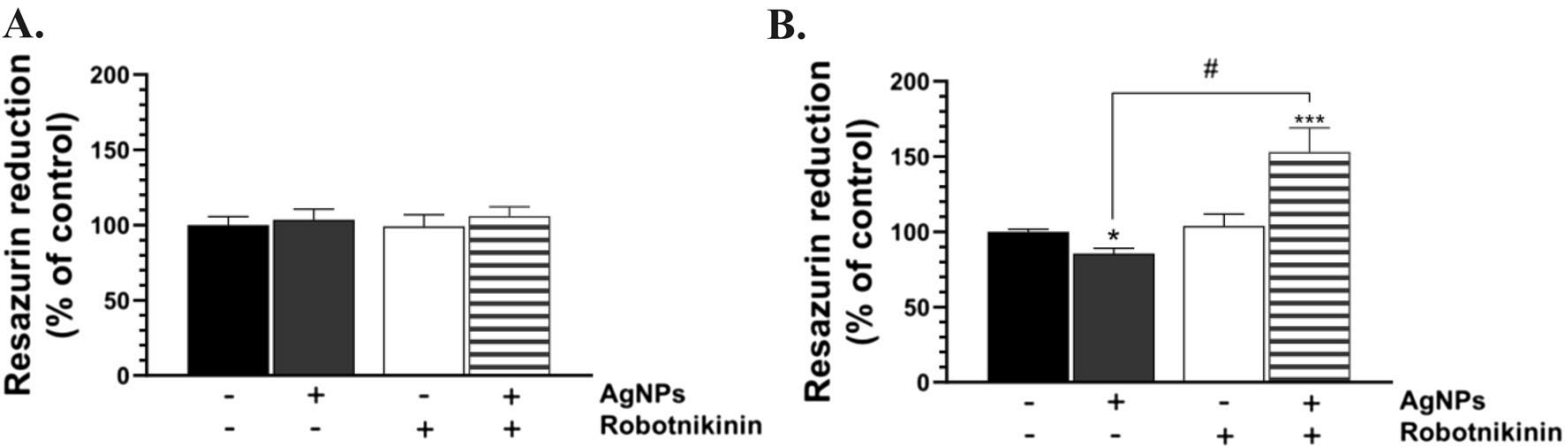
Metabolic activity of the U-87MG cells treated with 1 μg/mL of AgNPs, 10 μM of Robotnikinin, and/or co-treated with AgNPs/Robotnikinin for 24 h (**A**) and 48 h (**B**). The means ± SD denoted as *, **, and *** are statistically different from the control at *p* < 0.05, *p* < 0.001, and *p* < 0.001, respectively. #denotes statistical differences between certain groups at *p* < 0.05

The U-87MG cells treated with AgNPs, Robotnikinin, or co-treated with AgNPs/Robotnikinin were characterized by no significant changes in the metabolic activity after 24 h (Fig. 2A).

**Fig. 3:**
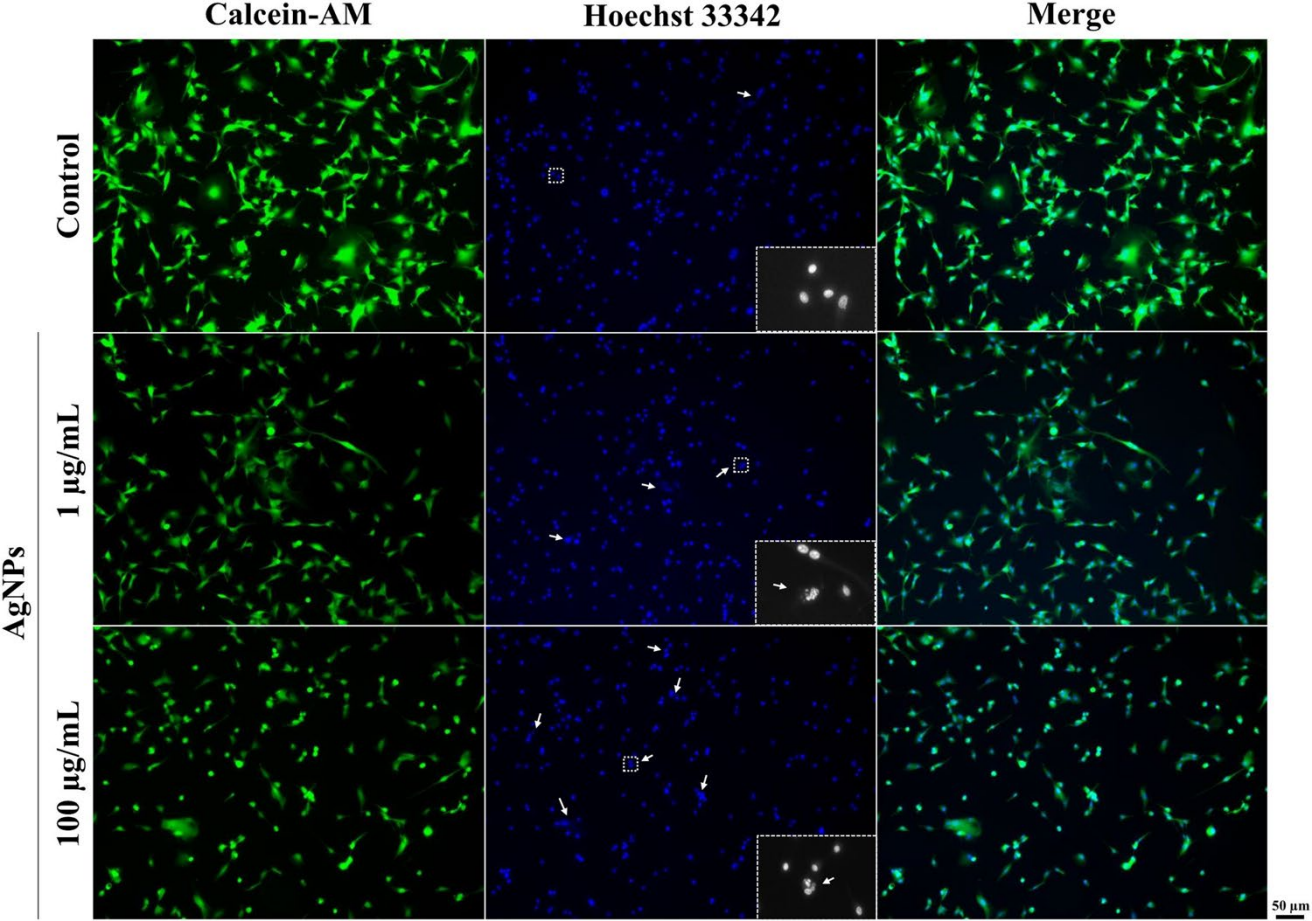
Effect of the Calcein-AM and Hoechst 33,342 staining of the U-87MG cells after the treatment with 1 µg/mL or 100 µg/mL for 48 h. The white arrows mark apoptotic nuclei. The image inserts show examples of normal (Control) and apoptotic nuclei (1 µg/mL or 100 µg/mL). The 100 × magnification was used

In turn, after 48 h, a 14.46% decrease in the metabolic activity was observed in the AgNP-treated cells, compared to the control, while the cells co-treated with AgNPs/Robotnikinin showed a 53.00% increase in this parameter, compared to the control (Fig. 2B). Additionally, a 67.46% statistically different effect between AgNPs- and AgNP/Robotnikinin-treated cells was observed (Fig. 2B). The apoptotic-like changes in nuclei after treatment with AgNPs have been observed (Fig. 3).

### *SHH* mRNA expression

**Fig. 4:**
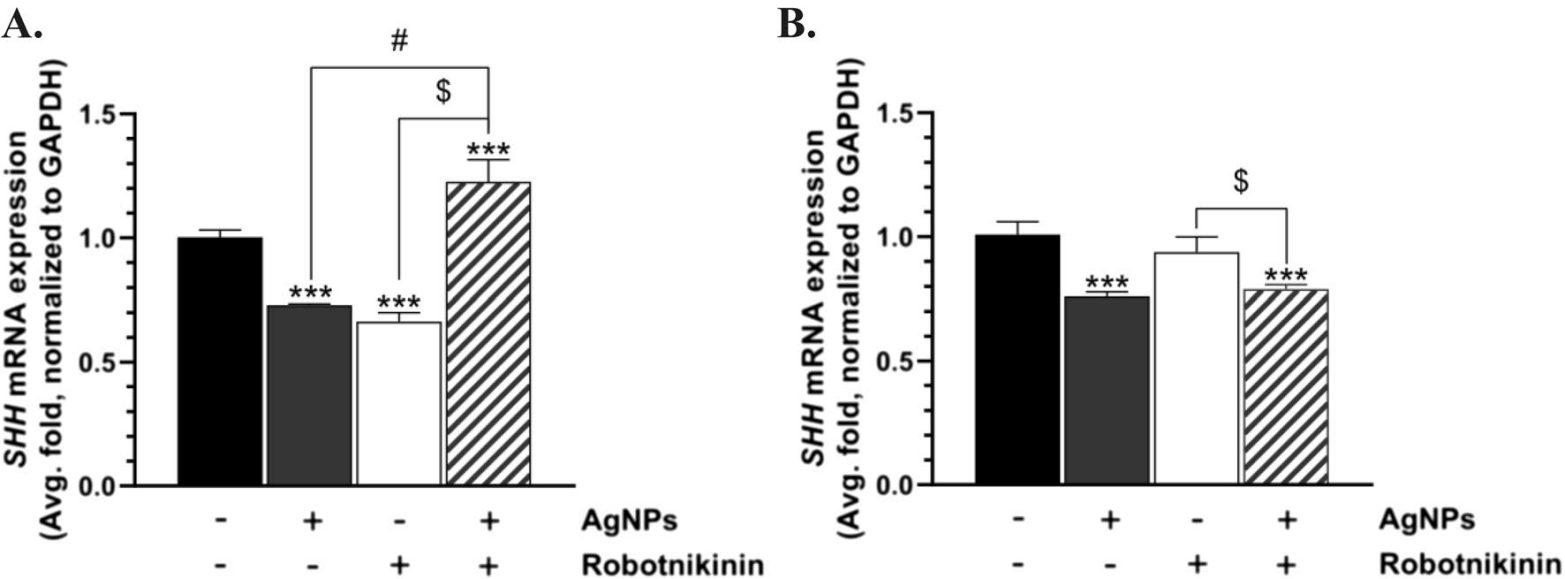
*SHH* mRNA expression in the U-87MG cells treated with AgNPs, Robotnikinin, and AgNPs/Robotnikinin for 6 h (**A**) and 24 h (**B**). The means ± SD (standard deviations) denoted as *, **, and *** are statistically different, compared to the control cells at *p* < 0.05, *p* < 0.01, and *p* < 0.001, respectively. The data marked as # or $ are statistically different between certain groups at *p* < 0.05

The U-87MG cells treated for 6 h with AgNPs and Robotnikinin were characterized by a 27.05% and 33.60% decrease in the *SHH* mRNA expression, respectively, compared to the control (Fig. 4A). In turn, a 22.61% increase in the expression of this gene was observed in cells treated with AgNPs/Robotnikinin, compared to the control (Fig. 4A). Moreover, a 49.69% statistically different *SHH* gene expression was observed in the AgNP-treated cells, compared to the AgNP/Robotnikinin-treated cells (Fig. 4B). After 24 h, a significant 24.06% and 21.13% decrease in the *SHH* mRNA expression was noted in the U-87MG cells treated with AgNPs and AgNPs/Robotnikinin, respectively, compared to the control (Fig. 4B). No significant changes in the expression of this gene were observed after the treatment of the tested cells with Robotnikinin (Fig. 4B).

### Protein expression level

**Fig. 5:**
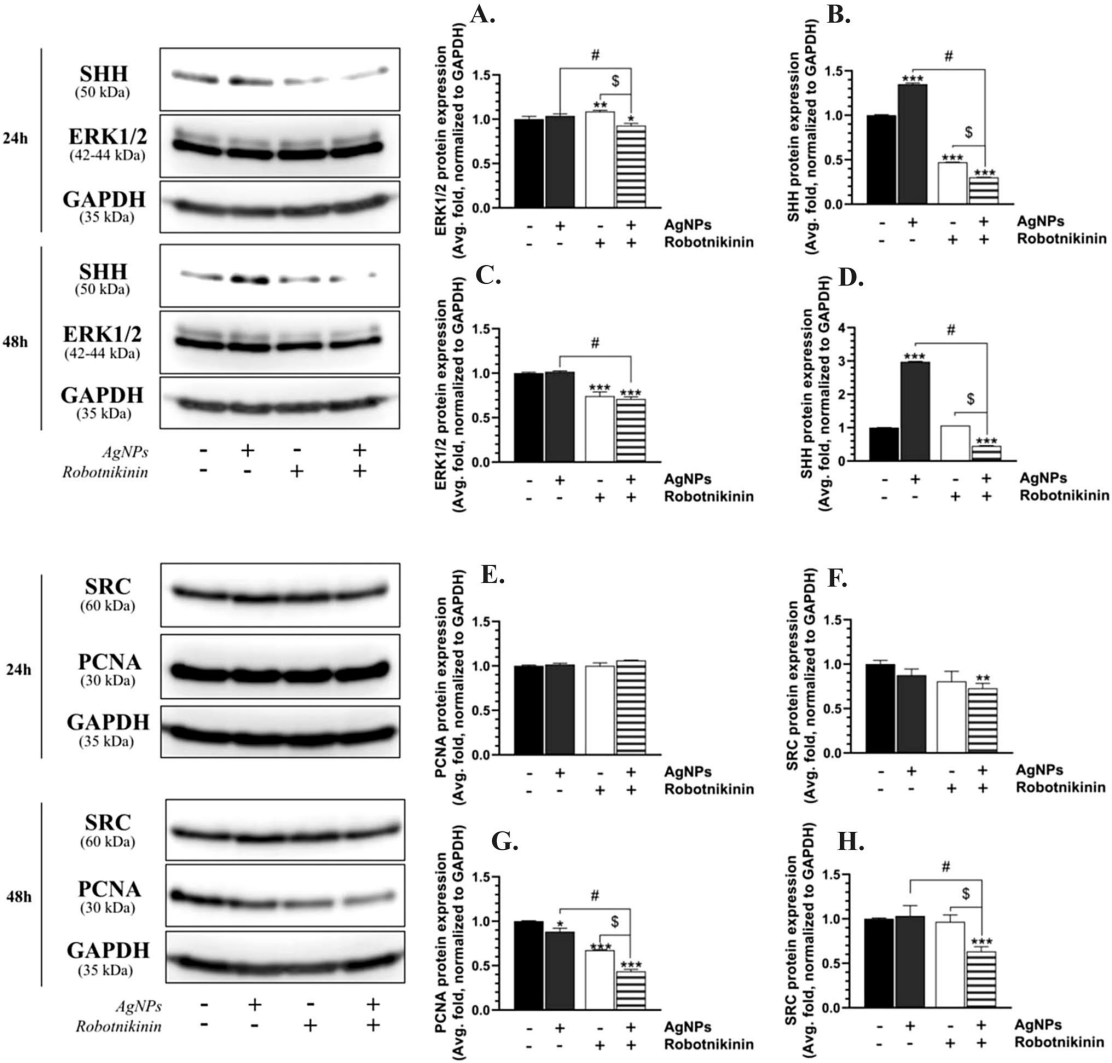
ERK1/2 (**A**, **C**), SHH (**B**, **D**), PCNA (**E**, **G**), and SRC (**F**, **H**) protein expression after the treatment of the U-87MG cells with 1 μg/mL of AgNPs, 10 μM of Robotnikinin, and AgNPs/Robotnikinin for 24 h (**A**, **C**, **E**, **G**) and 48 h (**B**, **D**, **F**, **H**). The means ± SD (standard deviations) denoted as *, **, and *** are statistically different, compared to the control cells at *p* < 0.05, *p* < 0.01, and *p* < 0.001, respectively. The data marked as # and $ are statistically different between certain groups at *p* < 0.05

After the 24-h U-87MG treatment with Robotnikinin, the ERK1/2 protein expression increased by 8.86%, compared to the control, while the cells co-treated with AgNPs/Robotnikinin showed a 7.19% decrease in the expression of this protein, compared to the control (Fig. 5A). Moreover, the effect on the AgNP/Robotnikinin-treated cells was statistically lower by 10.77%, compared to the AgNP-treated cells (Fig. 5A). In turn, after 48 h, the ERK1/2 protein expression in the U-87MG cells treated with Robotnikinin and AgNPs/Robotnikinin was decreased by 25.71% and 28.99%, respectively, compared to the control (Fig. 5C). The effect of the AgNP/Robotnikinin co-treatment on the ERK1/2 protein expression was 30.55% lower than in the AgNP-treated cells (Fig. 5C).

The U-87MG cells treated with AgNPs for 24 h were characterized by a 34.94% increase in the SHH protein expression, compared to the control (Fig. 5B). Conversely, 52.88% and 69.78% decrease in the expression of this protein was observed in the cells treated with Robotnikinin and AgNPs/Robotnikinin, respectively, compared to the control (Fig. 4B). A 104.74% statistically different effect between the AgNP- and AgNP/Robotnikinin-treated cells was noticed (Fig. 5B). After 48 h, the SHH protein expression increased by 197.62% in the AgNP-treated U-87MG cells, compared to the control, while the decrease in the AgNP/Robotnikinin-treated cells reached 54.68%, compared to the control (Fig. 5D). This effect differed by 252.30% from that in the AgNP-treated cells (Fig. 5D).

After 24 h, no significant changes in the PCNA protein expression were observed in the U-87MG cells (Fig. 5E). In turn, after 48 h, the cells treated with AgNPs, Robotnikinin, and AgNPs/Robotnikinin were characterized by an 11.80%, 32.69%, and 56.52% decrease in the PCNA protein expression, respectively, compared to the control (Fig. 5G). The effect of the AgNP treatment on the PCNA protein expression was statistically higher (by 44.72%) than in the AgNP/Robotnikinin-treated cells (Fig. 5G).

The U-87MG cells co-treated with AgNPs/Robotnikinin for 24 h were characterized by a 27.29% decrease in the SRC protein expression, compared to the control (Fig. 5F). In turn, the 48-h exposure of the tested cells to AgNPs/Robotnikinin decreased the expression of this protein by 36.62%, compared to the control (Fig. 5H). This effect differed by 39.91% from that in the AgNP-treated cells (Fig. 5H).

**Fig. 6:**
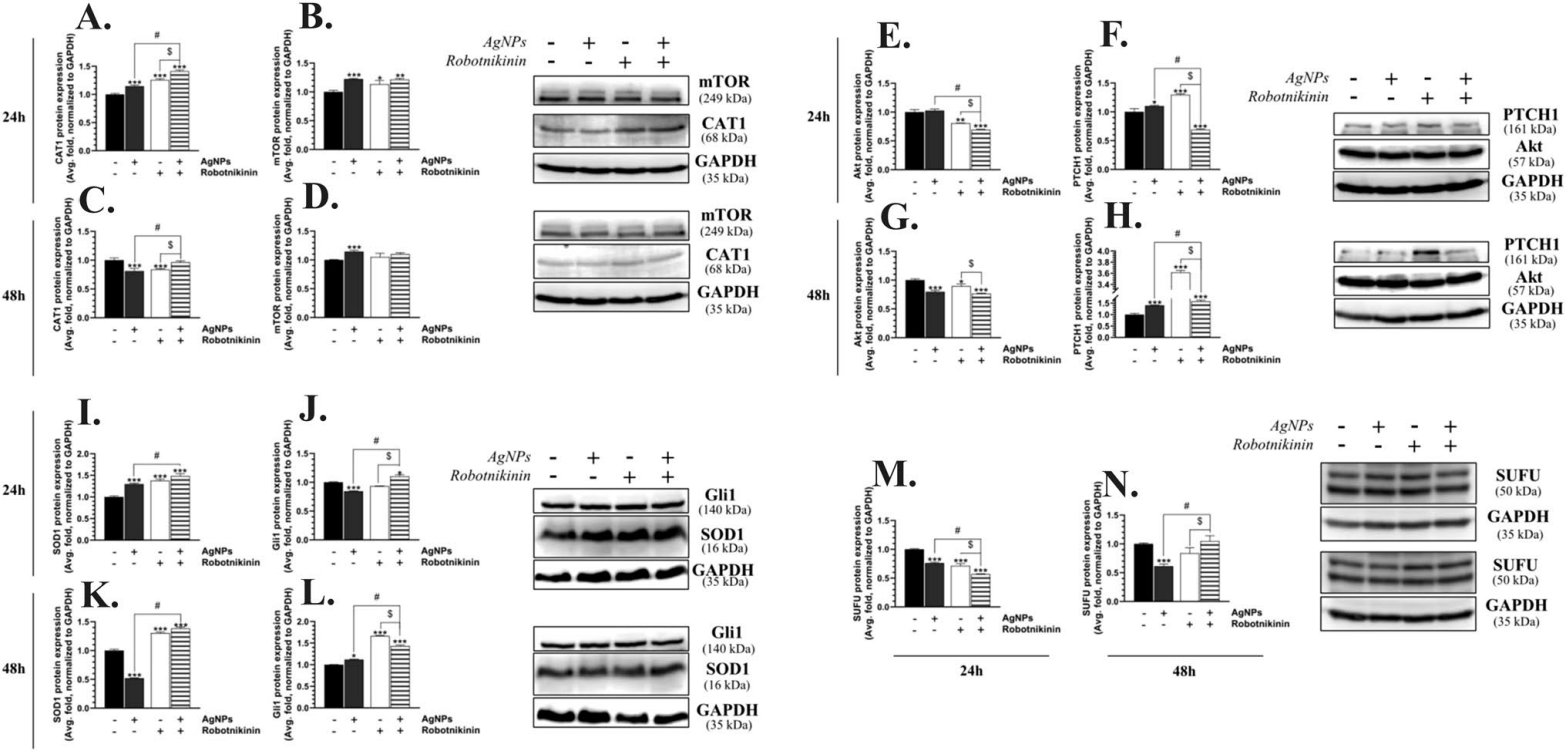
CAT1 (**A**, **C**), mTOR (**B**, **D**), Akt (**E**, **G**), PTCH1 (**F**, **H**), SOD1 (**I**, **K**), Gli1 (**J**, **L**), and SUFU (**M**, **N**) protein expression after the treatment of the U-87MG cells with 1 μg/mL of AgNPs, 10 μM of Robotnikinin, and AgNPs/Robotnikinin for 24 h (**A**,** B**,** E**,** F**,** I**,** J**,** M**) and 48 h (**C**, **D**,** G**,** H**,** K**,** L**,** N**). The means ± SD (standard deviations) denotes as *, **, and *** are statistically different, compared to the control cells at *p* < 0.05, *p* < 0.01, and *p* < 0.001, respectively. The data marked as # and $ are statistically different between certain groups at *p* < 0.05

The CAT1 protein expression after the treatment of the U-87MG cells with AgNPs, Robotnikinin, and AgNPs/Robotnikinin was significantly increased by 14.75%, 25.99%, and 41.33%, respectively, compared to the control (Fig. 6A). Moreover, a significant 26.58% increase in the expression of this protein was observed in the cells treated with AgNPs/Robotnikinin, compared to the AgNP-treated cells (Fig. 6A). In turn, after 48 h, the cells were characterized by an 18.90% and 16.25% decrease in the CAT1 protein expression in the variants with AgNPs and Robotnikinin, respectively (Fig. 6C). The observed effect in the AgNP-treated cells was statistically different (by 15.14%), compared to the AgNP/Robotnikinin-treated cells (Fig. 6C).

After 24 h, the U-87MG cells treated with AgNPs, Robotnikinin, and AgNPs/Robotnikinin were characterized by a 22.40%, 13.40%, and 21.43% increase in the mTOR protein expression, respectively, compared to the control (Fig. 6B). In turn, a statistically different 14.50% increase in the AgNP-treated cells was observed after 48 h, compared to the control (Fig. 6D).

The Akt protein expression was decreased by 19.17% and 30.51% in the Robotnikinin and AgNP/Robotnikinin-treated cells after 24 h, respectively, compared to the control (Fig. 6E). The effect of the AgNP/Robotnikinin co-treatment in the U-87MG cells was statistically different than in the AgNP-treated cells (33.19%) (Fig. 6E). In turn, after the 48-h treatment, the cells treated with AgNPs, Robotnikinin, and AgNPs/Robotnikinin were characterized by a 20.15%, 10.49%, and 23.24% decrease in the Akt protein expression, respectively, compared to the control (Fig. 6G).

After 24 h, the PTCH1 protein expression was increased by 10.03% and 29.68% in the U-87MG cells treated with AgNPs and Robotnikinin, respectively, compared to the control (Fig. 6F). In contrast, a 31.07% decrease in the expression of this protein was observed in the AgNP/Robotnikinin co-treated cells, compared to the control (Fig. 6F). This effect was by 41.11% different from that in the AgNP-treated cells (Fig. 6F). In turn, after the 48-h treatment of the cells with AgNPs, Robotnikinin, and AgNPs/Robotnikinin, the PTCH1 protein expression increased by 40.27%, 261.69%, and 58.06%, respectively, compared to the control (Fig. 6H). The effect caused by AgNPs was statistically lower (by 17.79%) than in the AgNP/Robotnikinin-treated cells (Fig. 6H).

The SOD1 protein expression was increased by 29.98%, 37.63%, and 48.33% in the AgNP-, Robotnikinin-, and AgNP/Robotnikinin-treated cells, respectively, compared to the control, after 24 h (Fig. 6I). Similarly, a 30.34% and 38.22% increase in the expression of this protein was observed in the cells treated with Robotnikinin and AgNPs/Robotnikinin, respectively, compared to the control, while a 47.96% decrease in the SOD1 protein expression in the AgNP-treated cells was observed, compared to the control, after 48 h (Fig. 6K). The effect caused by AgNPs and AgNPs/Robotnikinin differed by 86.18% (Fig. 6K).

After 24 h, the Gli1 protein expression in the U-87MG cells treated with AgNPs was decreased by 15.55%, compared to the control, while the AgNPs/Robotnikinin co-treatment caused a 10.42% increase in the expression of this protein, compared to the control (Fig. 6J). The observed effect was statistically different between these groups, and the difference was estimated at 25.97% (Fig. 6J). In turn, after 48 h, the Gli1 protein expression increased by 11.98%, 66.25%, and 43.36% in all the tested groups exposed to AgNPs, Robotnikinin, and AgNPs/Robotnikinin, respectively, compared to the control (Fig. 6L). Additionally, the effect caused by AgNPs and AgNPs/Robotnikinin differed between these two groups by 31.38% (Fig. 6L).

The SUFU protein expression after the 24-h treatment of the U-87MG cells with AgNPs, Robotnikinin, and AgNPs/Robotnikinin decreased by 23.76%, 28.54%, and 42.98%, respectively, compared to the control (Fig. 6 M). The effect between AgNPs and AgNP/Robotnikinin-treated cells differed by 19.23% (Fig. 6M). In turn, after the 48-h treatment, the cells treated with AgNPs were characterized by a 38.76% decrease in the SUFU protein expression, compared to the control (Fig. 6N). This effect was statistically different (by 43.75%) than in the AgNP/Robotnikinin-treated cells (Fig. 6N).

## Discussion

AgNPs are well-established cytotoxic factors, whose biological activity is strictly related to their high pro-oxidative properties leading to *redox* imbalance and, in consequence, oxidative stress (Maurer and Meyer 2016). The present results showed a significant increase in the intracellular ROS level only after 6 h of treatment, which was strengthened with the time of exposure (especially in the concentration range between 100 ng/mL and 1 µg/mL). These findings are similar to the results reported by Onodera et al., who proved the ability of 1-nm AgNPs applied at the 5 µg/mL concentration to increase the ROS level in BALB/3T3 A31-1–1 cells (Onodera et al. 2015). Furthermore, Kang et al. showed that exposure to a low 2 µg/mL concentration of AgNPs (2.3 nm) significantly increased ROS production in the murine dendritic cell line (DC2.4) (Kang et al. 2012). In turn, in our study, a massive decrease in the ROS production level was observed in some of the tested concentrations of AgNPs—10 µg/mL, 50 µg/mL, and 100 µg/mL, which was presumably caused by their high cytotoxic effect. Indeed, the resazurin reduction assay showed that, in the microgram concentrations, AgNPs were able to decrease the metabolic activity by approx. 50%, resulting in IC_50_ values of 30.41 µg/mL and 21.16 µg/mL in the 24-h and 48-h AgNP exposure variants, respectively. Moreover, our further studies indicated a decrease in the PCNA protein expression (well-established proliferation marker) after 48 h in the U-87MG treated with AgNPs, which proves the aforementioned phenomenon. Similarly, our previous study showed that small-size AgNPs were able to decrease KI67 protein expression in human normal fibroblasts (BJ), lung adenocarcinoma cells (A549), and squamous tongue carcinoma (SCC-15) (Skóra et al. 2022). Moreover, Chairuangkitti et al. proved that AgNPs (100 nm) were able to arrest A549 cells in the sub-G1 cell cycle phase with a decrease in PCNA protein expression, which is associated with the ROS-mediated pathway (Chairuangkitti et al. 2013). The toxicity of AgNPs was shown *inter alia* by Liang et al. in U-87MG cells and by Salazar-García et al. in rat GB cells (C6) (Liang et al. 2017; Salazar-García et al. 2020). Additionally, our results of confocal microscopy analysis showed the ability of tested AgNPs to induce apoptotic-like changes in the nucleus in tested cell line. Therefore, the results obtained in this study are consistent with the current state of knowledge and show the high anti-cancer potential of AgNPs to be used in GB therapy; however, this has never been tested in the context of the HH pathway.

It has been reported that ROS may affect the SHH–NOX4–HIF1α pathway in cerebellar progenitor cells (Eyrich et al. 2019). Therefore, Robotnikinin was used in the next part of this study. This compound has been identified as a selective inhibitor of SHH, whose mechanism of action is related to the blocking of the ability of SHH to bind to PTCH1 (Stanton and Peng 2010). The co-treatment of the U-87MG cells with AgNPs and Robotnikinin abolished the cytotoxic effect of AgNPs, which may suggest some correlation between the SHH pathway and the mechanism of action of AgNPs. However, due to the rather overall non-specific character of the resazurin reduction assay, the *SHH* mRNA expression was assessed. Our data showed that AgNPs had an impact on the *SHH* gene expression in a similar way as Robotnikinin, reducing the mRNA expression of the gene. A massive decrease in the expression of certain genes is often correlated with an increase in the expression of the corresponding protein, which in turn acts as a negative regulator of the expression of this gene (Ivanov 2019). Indeed, the measured SHH protein expression showed an increase in this parameter in both time intervals after the treatment with AgNPs. As cited above, AgNPs act mainly by inducing high amounts of ROS, which is consistent with results reported by Dai et al., who showed activation of the SHH pathway in H_2_O_2_-treated primary rat cortical neurons, resulting in a time-dependent increase in the SHH protein expression as part of the protective mechanism (Dai et al. 2011). On the other hand, Kim et al. concluded that oxidative stress inhibited SHH-induced osteogenic differentiation of multipotent bone marrow stromal cells (Kim et al. 2010). Therefore, it can be hypothesized that AgNPs exert the inhibitory effect on the SHH pathway in a ROS-dependent manner. Interestingly, in our study, Robotnikinin and/or AgNPs/Robotnikinin showed an opposite effect, i.e., a decrease in the SHH protein expression, which may suggest a different or excluding mechanism of action between these two compounds.

The predicted ROS-dependent mechanism of AgNPs and its effect on the SHH pathway is consistent with the results of the SOD1 and CAT1 protein expression, which are well-established markers of oxidative stress (Patlolla et al. 2009). After the treatment of the U-87MG cells with AgNPs for 24 h, the SOD1 and CAT1 protein expression was significantly increased, followed by a decrease in the expression of these proteins after 48 h, which indicated the ability of the tested NPs to induce oxidative stress. A similar tendency was shown for Robotnikinin. Although the pro-oxidative properties of AgNPs in GB (DBTRG-05MG) cells were shown *inter alia* by Akyuva and Nazıroğlu, the direct effect of Robotnikinin on oxidative stress induction has never been tested (Akyuva and Nazıroğlu 2023). However, recently (2022), Karadağ and Başbinar have shown an inhibitory effect of Robotnikinin and Vismodegib (SHH inhibitor at the SMO level) on U-87MG proliferation and invasiveness, which is consistent with the results shown in this study (Karadağ and Basbinar 2022). Nevertheless, to investigate whether AgNPs affect the canonical or non-canonical HH pathway, the expression of specific downstream proteins was measured.

As shown by the literature data, the canonical HH pathway is based on the mutual interaction between PTCH1, SMO, SUFU, and Gli-1 proteins (Teperino et al. 2014). Both Gli1 and PTCH1 play a crucial role in the HH pathway, which is related to the binding of SHH to PTCH1 and transducing this signal intracellularly with Gli1, upregulating certain pro-proliferative genes (Cohen et al. 2015). Interestingly, Ji et al. have proved that the inhibition of the SHH pathway by cyclopamine (at the SMO level) results in increased DNA damage and downregulation of certain downstream proteins in the SHH pathway, *inter alia* Gli1 and PTCH1 (Ji et al. 2012). Similar data were shown in our study. The tested AgNPs were able to decrease the Gli1 protein expression (transcriptional factor of the SHH pathway) after 24 h, in contrast to Robotnikinin, which did not affect this parameter. Nevertheless, an inverse effect was observed in the co-treated cells. Moreover, the PTCH1 protein expression was significantly increased after the treatment with AgNPs for 24 h and 48 h. Furthermore, this effect was statistically different after the co-treatment of the U-87MG cells with AgNPs and Robotnikinin. These results are consistent with those reported by Wang et al., who showed the ability of cyclopamine to decrease the *PTCH1, SMO*, and *Gli1* mRNA expression in human pancreatic cancer (PANC-1) and human colon cancer (HT-29, LoVo, and HCT-116) cell lines in vitro (Wang et al. 2014). On the other hand, some SHH inhibitors seem to act in a cell-specific manner, which was confirmed by the results obtained by Carballo et al., who proved an effect of cyclopamine on PTCH1 protein expression in GBM95 cells but not in GBM02 and GBM03 cells (Carballo et al. 2020). Interestingly, it was shown in the present study that AgNPs were able to decrease the SUFU protein expression in both tested time intervals. SUFU is classified as a regulator of the Gli1-dependent pro-proliferative ability of the HH pathway (Yan et al. 2021). The decrease in SUFU and Gli1 presented in this study proved the cytotoxic properties of AgNPs, shown *inter alia* by the PCNA protein expression and by the resazurin reduction assay, and are consistent with the results reported by Wang et al. and Liu et al. (Liu et al. 2018a; Wang et al. 2013). However, this study is the first to show that AgNPs can reduce the proliferation of U-87MG cells with engagement of the Gli1 transcriptional factor. Moreover, the results shown above also prove the ability of AgNPs to block the SHH pathway first and later decrease the downstream effectors of this pathway-related protein, leading to a decrease in the *SHH* mRNA expression, probably by blocking the PTCH1 ability to bind SHH. These results are crucial, given the recent findings of the overexpression of SHH and/or Gli1 in GBs and other cancers, leading to lower survival rates in patients (Budimir et al. 2022; Cui et al. 2010).

It has been reported that the HH pathway can also be activated as a result of the activity of certain kinases, *inter alia* ERK1/2, Akt, and/or mTOR, i.e., the non-canonical pathway (Wang et al. 2012). The results obtained in this study showed that the tested small-size AgNPs did not affect the ERK1/2 protein expression in any time interval, in contrast to Robotnikinin, which alone and in the co-treatment with the AgNPs reduced the expression of this protein. This is consistent with the results shown by Liu et al., who proved that cyclopamine decreased the proliferation of fibroblast-like synoviocytes by reducing ERK1/2 phosphorylation (Liu et al. 2018b). This also suggests that AgNPs exert an anti-proliferative effect on GBs cells independently of ERK1/2, but rather with engagement of the canonical HH pathway. This is opposite to the results reported by Castiglioni et al., who discovered that the engagement of ERK1/2 and its phosphorylation state were crucial for induction of AgNP cytotoxicity in bladder carcinoma cells (T24) (Castiglioni et al. 2015). Similarly, Rinna et al. showed that, in addition to oxidative stress, AgNPs were able to induce DNA damage, which was strengthened after ERK1/2 blocking in human epithelial embryonic cells (Rinna et al. 2015). The differences between the cited papers and the present results may be an effect of the different cell model used in the studies as well as the higher diameter of AgNPs (20 nm and 35 nm vs. 5 nm tested in this study).

**Fig. 7:**
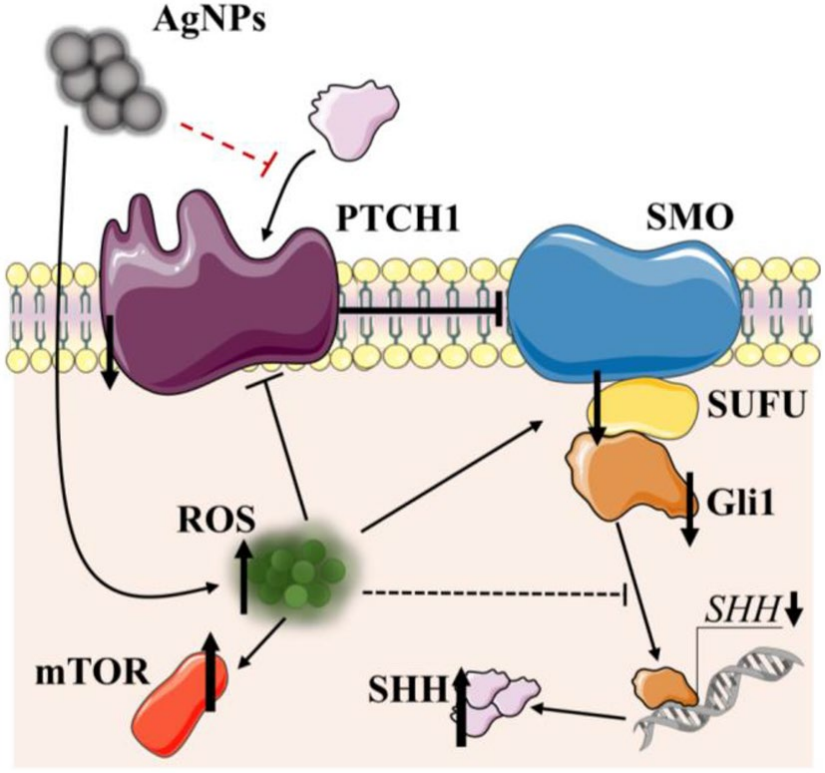
Proposed mechanism of action of AgNPs in U-87MG cells. After the uptake, AgNPs cause an increase in the cellular ROS level, which subsequently affects the PTCH1 receptor, resulting in a decrease in the ability to bind SHH. Consequently, U-87MG cells are characterized by overexpression of the SHH protein (compensation effect), resulting in a decrease in the *SHH* mRNA expression (probably by negative feedback). In consequence, the HH downstream proteins (SMO, SUFU, and Gli1) are downregulated, resulting in reduced U-87MG proliferation

Interestingly, as shown by Katoh in 2009, Gli1 can be activated by the PI3K/Akt pathway, which is related to the ability of Akt to stabilize Gli1 (Katoh 2009). In our study, the tested AgNPs did not exert an impact on the Akt protein expression after the 24-h treatment, in contrast to the effect caused by Robotnikinin. Therefore, it is justified to assume that AgNPs inhibit the proliferation of GBs based via the canonical rather than non-canonical pathway, which supports the hypothesis of the different mechanisms of action between AgNPs and Robotnikinin. Indeed, Adnan et al. demonstrated that small-size AgNPs (6–20 nm) were able to be uptaken in U-251 cells after only 2 h, which proved their ability to induce oxidative stress in a short-time treatment, as shown in this study as well (Adnan et al. 2020). Because our data did not show a decrease in the Akt protein expression 24 h after the AgNP treatment but the impact of the nanoparticles on the *SHH* mRNA expression was observed after only 6 h, followed by the massive increase in the SHH protein expression, it is justified to assume that the impact of AgNPs on the HH pathway is ERK1/2- and Akt-independent. The further decrease in the Akt protein expression was probably an effect of the AgNP- and Gli1-dependent cytotoxicity. This is consistent with the results shown by Elekofehinti, who proved that AgNPs were able to decrease *AKT* mRNA protein expression in Wistar rats (Elekofehinti et al. 2021). Moreover, the present results showed an increase in the mTOR protein expression after the treatment of the U-87MG cells with AgNPs, which is consistent with the results reported in the literature *inter alia* by Li et al. in neuroblastoma cells (SH-SY5Y) or by Chang et al. in mouse hippocampal neurons (HT-22), suggesting the potential of the tested AgNPs to induce autophagy (Li et al. 2019; Chang et al. 2021). However, taking the above results and the cited studies into account, this study is the first to show that small-size AgNPs (5 nm) are able to act as inhibitors of the HH pathway, probably via an ROS-dependent pathway. These interactions subsequently lead to a reduced PTCH1 ability to bind SHH, which results in the suppression of the downstream proteins of the HH pathway, including the Gli1 transcriptional factor. Moreover, the non-canonical activation of HH is not likely to be engaged in the AgNP mechanism of action. The present results also prove the postulated suitability of these NPs in the GB treatment, especially in the context of the potential SHH role in the MDR phenomenon in such cells. Based on the results, the proposed mechanism of tested NPs' action were shown below (Fig. 7). However, more comprehensive studies are needed to fully elucidate the AgNPs’ role in this field.

## Conclusions

The study shows for the first time that AgNPs are able to suppress the HH pathway at the mRNA and protein expression level based on the ROS-dependent pathway in GBs cells. The tested AgNPs were able to decrease the ability of SHH to bind to PTCH1 and downregulate the downstream proteins related to the canonical HH pathway, which resulted in a decrease in the proliferation of the U-87MG cells through suppression of SUFU-Gli1. Moreover, the observed effect was not correlated with the non-canonical HH pathway. The present results prove the potential suitability of small-size AgNPs in anti-cancer therapy, especially in the case of the recently proved Gli1 overexpression in GB cells.

## Data Availability

The data are available from the corresponding author on reasonable request.

## Abbreviations

ABC: ATP-binding cassette transporter
AgNPs: Silver nanoparticles
Ag^+^: Silver ions
BBB: Blood–brain barrier
DHH: Desert hedgehog
FBS: Fetal bovine serum
GBs: Glioblastomas
H_2_DCF-DA: 2′,7′-Dichlorodihydrofluorescein diacetate
HH: Hedgehog
IHH: Indian hedgehog
NRF2: Nuclear Factor Erythroid 2 Like 2
Robotnikinin: N-[(4-chlorophenyl)methyl]-5,12-dioxo-2R-phenyl-1-oxa-4-azacyclododec-8E-ene-6S-acetamide
PCNA: Proliferating cell nuclear antigen
PTCH1: Protein patched homolog 1
ROS: Reactive oxygen species
SHH: Sonic hedgehog
SMO: Smoothened protein
SUFU: Suppressor of fused homolog protein
